# SB202190 inhibits endothelial cell apoptosis via induction of autophagy and heme oxygenase-1

**DOI:** 10.18632/oncotarget.25234

**Published:** 2018-05-01

**Authors:** Margit Schwartz, Sabine Böckmann, Philipp Borchert, Burkhard Hinz

**Affiliations:** ^1^ Institute of Pharmacology and Toxicology, Rostock University Medical Center, Rostock, Germany

**Keywords:** SB202190, p38 MAPK, heme oxygenase-1, apoptosis, autophagy

## Abstract

Activation of the p38 mitogen-activated protein kinase (MAPK) pathway has been implicated in various detrimental events finally leading to endothelial dysfunction. The present study therefore investigates the impact of the p38 MAPK inhibitor SB202190 on the expression of the cytoprotective enzyme heme oxygenase-1 (HO-1) as well as metabolic activity, apoptosis and autophagy of endothelial cells. Using human umbilical vein endothelial cells (HUVEC) SB202190 was found to cause a time- and concentration-dependent induction of HO-1 protein. Induction of HO-1 protein expression was mimicked by SB203580, another p38 MAPK inhibitor, but not by SB202474, an inactive structural analogue of p38 MAPK inhibitors. HO-1 induction by both SB202190 and SB203580 was also demonstrated by analysis of mRNA expression. On the functional level, SB202190 was shown to increase metabolic activity and autophagy of HUVEC along with diminishing basal apoptosis. Treatment of cells with tin protoporphyrin IX (SnPPIX), a well-characterised HO-1 enzymatic inhibitor, or HO-1 siRNA left SB202190-modulated metabolic activity and autophagy virtually unaltered but caused a significant reversal of the anti-apoptotic action of SB202190. Conversely, however, HO-1 expression by SB202190 became completely suppressed by the autophagy inhibitor bafilomycin A_1_. Bafilomycin A_1_ likewise fully reversed effects of SB202190 on metabolic activity and apoptosis, albeit significantly inducing apoptosis per se. Collectively, this work demonstrates SB202190 to confer upstream induction of autophagy followed by HO-1 induction resulting in potential protective effects against apoptosis. On the other hand, our data oppose HO-1 to contribute to SB202190-mediated increases in metabolic activity and autophagy, respectively.

## INTRODUCTION

Inhibition of endothelial cell apoptosis has been associated with protective effects against the progression of inflammatory vascular diseases including atherosclerosis [1–3], which is the main source of pathological cardiovascular events, such as heart disease and stroke [4]. Inflammatory responses of the endothelium and vascular dysfunctions are often triggered by an imbalance between pro-oxidative and anti-oxidative mediators [5]. Among the latter group, the cytoprotective enzyme heme oxygenase (HO), consisting of an inducible HO-1 and a constitutive HO-2 isoform, has been attracted considerable attention in past years [5, 6]. The HO system regulates cellular heme homeostasis via rate-limitation of heme breakdown [7], thereby removing the potential cytotoxic molecule heme [8, 9] and producing equimolar amounts of potential cytoprotective biliverdin and carbon monoxide (CO) [5, 7, 10, 11].

As a critical negative regulator of endothelial cell function, the p38 mitogen-activated protein kinase (MAPK) has been described to modulate cell survival, migration and vascular permeability [12–16]. P38 MAPK has been implicated in the pathogenesis of atherosclerosis (for review see [17]). Pyridinyl imidazole compounds, originally developed as a novel class of cytokine biosynthesis inhibitors [18–20] and subsequently found to exert a specific inhibition of p38 α/β MAPK activity [21–26], have been described to reduce apoptosis of endothelial cells [27, 28] and myocytes [29] accordingly. In preclinical animal studies, p38 MAPK inhibitors have been reported to reduce atherosclerotic disease progression [16] and to protect against ischemic myocardial injury [30]. On the clinical level, the p38 MAPK inhibitor losmapimod has been shown to improve nitric oxide-mediated vasodilatation in hypercholesterolemic patients [31], to reduce vascular inflammation in the most inflamed regions in patients with atherosclerosis, concurrent with a reduction of inflammatory biomarkers [32], and to reduce circulating inflammatory markers as well as a marker of wall stress in patients with myocardial infarction [33]. In a recent trial, however, use of losmapimod compared with placebo did not reduce the risk of major ischemic cardiovascular events of patients with acute myocardial infarction [34]. To overcome the limitations of this study, the initiation of a prolonged and more investigative trial with a larger sample size has been recently suggested [35].

Notwithstanding the increasing knowledge on vasculoprotective effects of p38 MAPK inhibitors, the exact mechanism underlying the endothelial protective action of this group of substances is not completely understood. Thus, HO-1 should be addressed as possible target in view of data showing induction of HO-1 by p38 MAPK inhibition in macrophages and fibroblasts [36, 37]. Moreover, recent studies have associated p38 MAPK signalling with cellular autophagy [38–42], a catabolic process involving degradation of cellular material that maintains key functions of endothelial cell survival and enables adaptation to stress by recycling of energy stores (reviewed in [43, 44]). Other investigations presented evidence for a link between HO-1 and induction of autophagy [45, 46]. Collectively, this data led us to consider a possible mechanistic association of p38 MAPK inhibition, HO-1 and autophagy in endothelial cells. Using human umbilical vein endothelial cells (HUVEC), the present study therefore investigates a potential coordinated action within effects of the p38 MAPK inhibitor SB202190 on HO-1 expression, cellular autophagy and apoptosis. Here we present evidence for a hitherto unknown p38 MAPK inhibitor-elicited pathway involving an autophagy-dependent induction of HO-1 expression and resulting in HO-1-mediated inhibition of cellular apoptosis.

## RESULTS

### p38 MAPK inhibitors induce HO-1 protein and mRNA expression in HUVEC

**Figure 1:**
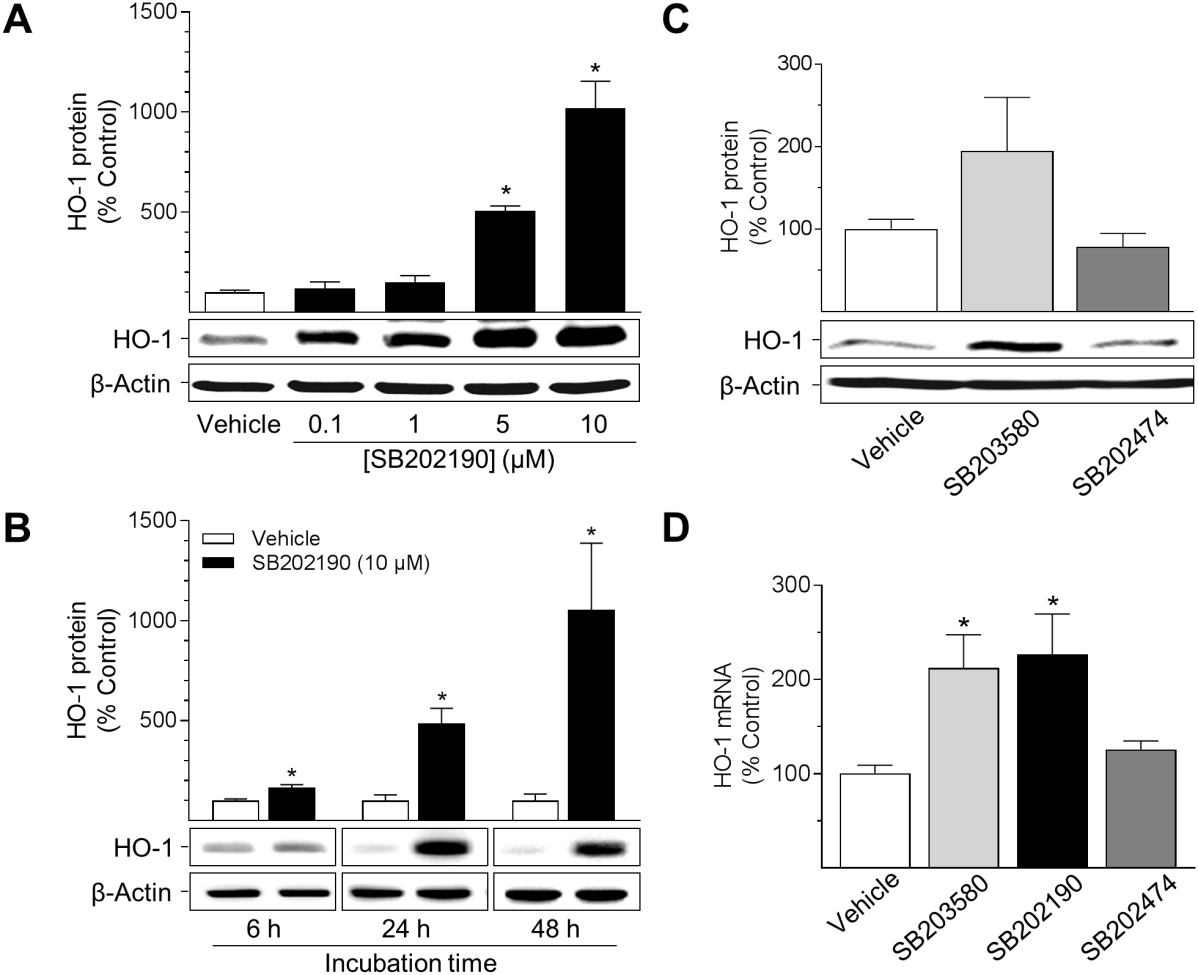
Effect of p38 MAPK inhibitors on HO-1 protein and mRNA expression in HUVEC Cells were incubated with p38 MAPK inhibitors (SB202190, SB203580) or inactive structural analogue SB202474 at indicated concentrations **(A)** or at 10 μM **(B, C, D)** for 24 h (A, C, D) or as indicated (B). After incubation, cells were analysed for protein or mRNA expression of HO-1. Protein and mRNA expression values were normalised to β-actin. Percent control represents comparison with the respective vehicle-treated time-matched group (set as 100%). Values are means ± SEM of n = 3 (A), n = 5 (B, C) or n = 5–6 (D) experiments. ^*^P < 0.05 vs. time-matched vehicle control; one-way ANOVA plus post hoc Dunnett test (A, C, D) or Student’s two-tailed *t* test (B).

Two different p38 MAPK inhibitors were analysed for their potential to induce the expression of HO-1 in HUVEC (Figure 1). SB202474, an inactive structural analogue, served as a negative control of p38 MAPK inhibition. SB202190 significantly increased HO-1 protein expression in a concentration- and time-dependent manner up to 10-fold (Figure 1A, 1B). At 10 μM, HO-1 protein expression became significant after 6 h (1.6-fold increase) and was further raised with prolonged incubation time (Figure 1B; 4.8-fold at 24 h; 10-fold at 48 h). The second p38 MAPK inhibitor, SB203580, increased HO-1 protein up to 1.9-fold after a 24-h incubation (Figure 1C). Further analyses revealed both SB202190 and SB203580 to increase HO-1 mRNA levels in HUVEC (Figure 1D). In contrast, incubation of cells with the inactive structural analogue SB202474 did not induce either HO-1 protein or HO-1 mRNA expression (Figure 1C, 1D). Due to the potent induction of HO-1 protein and mRNA, SB202190 was chosen for further experiments.

### SB202190 induces metabolic activity, suppression of apoptosis and G_0_/G_1_ cell cycle arrest in HUVEC

Metabolic activity of HUVEC was investigated as a parameter of cellular conditions. In parallel, DNA fragmentation of cells, indicating apoptosis, was analysed to evaluate potential effects on cell viability.

**Figure 2:**
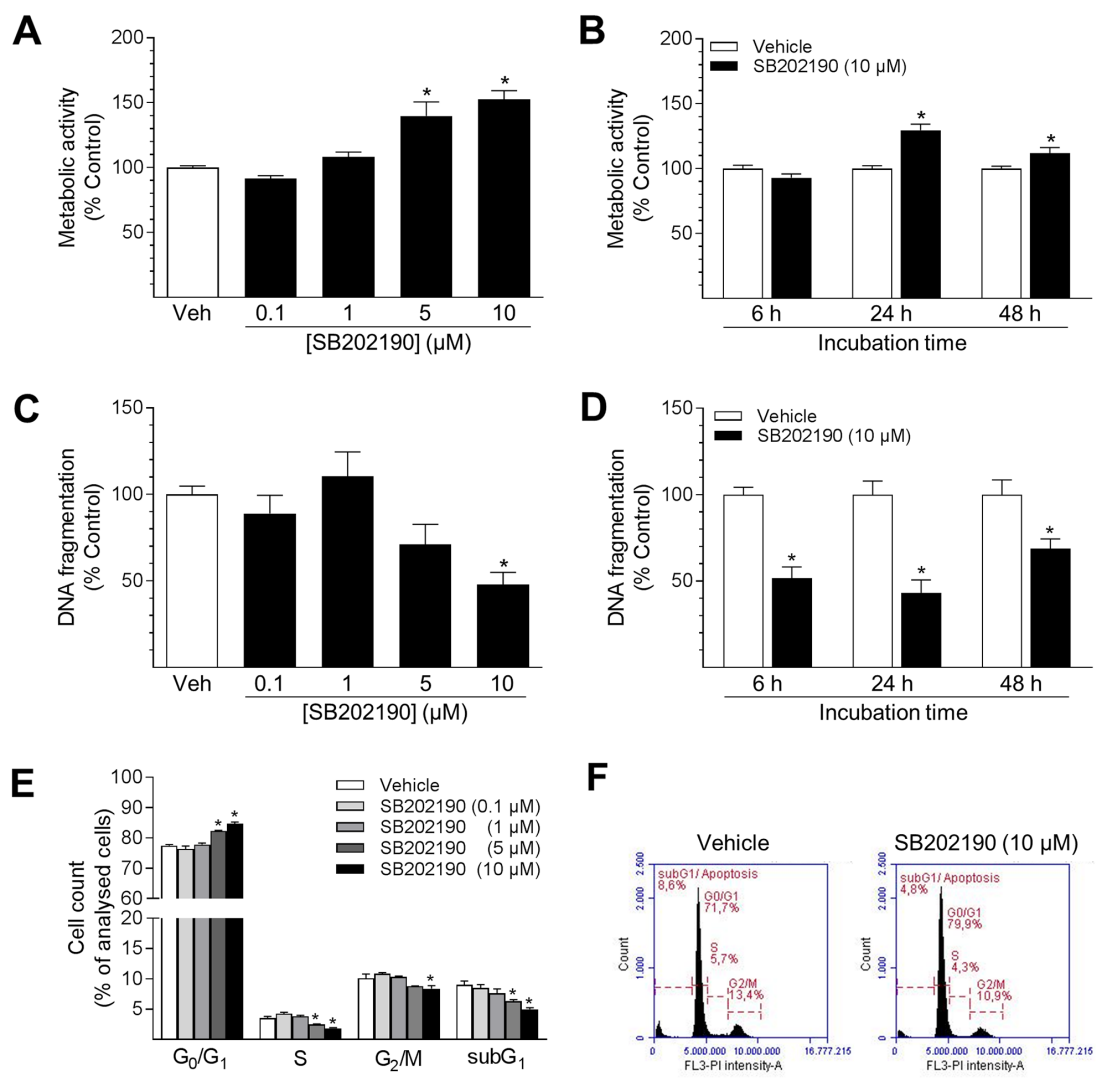
Impact of SB202190 on metabolic activity, apoptosis and cell cycle progression of HUVEC Cells were incubated with increasing concentrations of SB202190 or vehicle control for 24 h **(A, C, E)** or with 10 μM SB202190 for the indicated times **(B, D)**. Following incubation, cells were analysed for metabolic activity using WST-1 colorimetric assay (A, B), DNA fragmentation (C, D) or cell cycle distribution using flow cytometry (E). Exemplary images of flow cytometry analysis are shown for vehicle control and 10 μM SB202190, respectively **(F)**. Percent control represents comparison with the respective vehicle-treated time-matched group. Values are means ± SEM of n = 15–18 (A), n = 19–22 (B), n = 7–8 (C), n = 11–12 (D) and n = 3 (E) experiments. ^*^P < 0.05 vs. time-matched vehicle control; one-way ANOVA plus post hoc Dunnett test (A, C, E) or Student’s two-tailed *t* test (B, D).

As shown in Figure 2A and 2B, SB202190 enhanced the metabolic activity of cells in a concentration- and time-dependent manner. After 24 h, metabolic activity was significantly increased by 1.4- to 1.5-fold with 5 μM and 10 μM SB202190, respectively (Figure 2A). Kinetic studies revealed the process to be time-dependent: metabolic activity was not altered after 6 h, peaked after 24 h with a 1.3-fold increase and remained significant until a 48-h incubation with SB202190 (Figure 2B). However, metabolic activity was not altered when cells were treated with the inactive structural analogue SB202474: SB202474 (10 μM), 107% ± 3.7% vs. vehicle control (100% ± 3.9%), means ± SEM of n = 12–18.

To further investigate the effects of SB202190 on cellular functions, experiments analysing basal apoptosis were performed (Figure 2C, 2D). Following a 24-h incubation with 10 μM SB202190, detectable DNA fragmentation in HUVEC was significantly reduced to 48% vs. vehicle control (Figure 2C). In kinetic studies using 10 μM SB202190, DNA fragmentation of HUVEC was significantly diminished at all times analysed (Figure 2D). Maximal suppression of apoptosis was detected after a 24-h incubation with SB202190 (Figure 2D). In contrast, DNA fragmentation was not significantly altered when cells were treated with the inactive structural analogue SB202474: SB202474 (10 μM) 112% ± 37% vs. vehicle control (100% ± 17.3%), means ± SEM of n = 4.

Subsequent flow cytometry analyses confirmed the anti-apoptotic effect of SB202190 and revealed a shift of cell cycle progression in HUVEC (Figure 2E, 2F). Following a 24-h incubation, the subG_1_ population, indicating apoptotic cells, was decreased by SB202190 in a concentration-dependent manner (Figure 2E). At a final concentration of 10 μM, SB202190 was shown to decrease basal apoptosis of cells by 45%: SB202190 (10 μM) 5.00% ± 0.23% vs. vehicle control (9.02% ± 0.62%), means ± SEM of n = 3, % of analysed cell population (Figure 2E, 2F). Furthermore, the number of cells in the S phase was significantly decreased, indicating a diminished proliferation rate of cells after treatment with 5 and 10 μM SB202190. Reduction of both subG_1_ and S phase was accompanied by a concentration-dependent reduction of cells in the G_2_/M phase and a corresponding increase in the G_0_/G_1_ phase, respectively (Figure 2E).

### SB202190 mediates activation of autophagy processes in HUVEC

Autophagy has been described as a metabolically active process that supports the maintenance of basal energy balance under stressful conditions [43]. Therefore, activation of autophagy may explain the increase in metabolic activity of cells. Initiation of autophagy requires conjugation of microtubule-associated protein 1 light chain 3 I (LC3-I) to phosphatidylethanolamine (PE), generating LC3-II, for the maturation of autophagosomes [47, 48]. With respect to the literature referring to differential affinities of antibodies to LC3-I and LC3-II, as well as different expression levels of these proteins depending on cell line and tissue, activation of autophagy was quantified using the expression of PE-conjugated LC3A/B-II protein normalised to β-actin instead of the protein ratio between LC3-I and LC3-II [48, 49].

**Figure 3:**
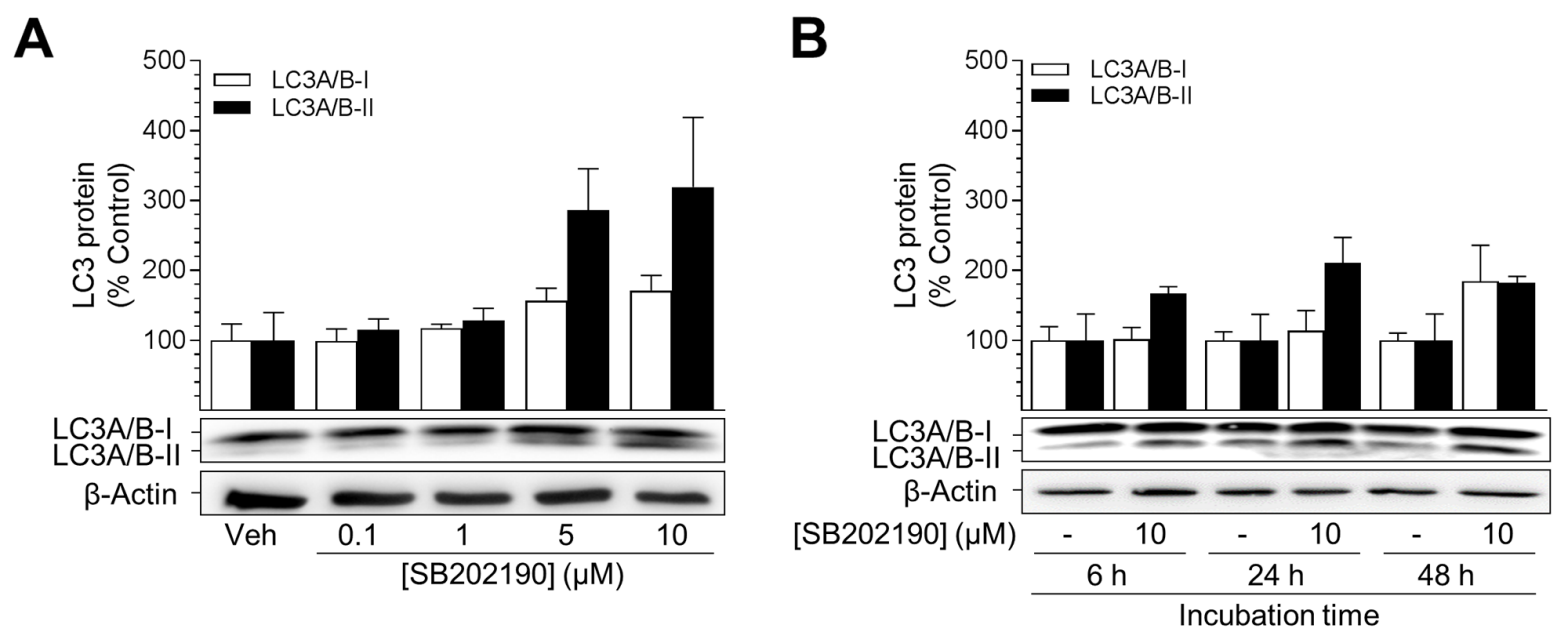
SB202190 triggers activation of autophagy in HUVEC Cells were incubated with increasing concentrations of SB202190 or vehicle control for 24 h **(A)** or with 10 μM SB202190 for the indicated times **(B)**. Following incubation, cells were harvested and lysates were analysed for autophagy-related protein LC3A/B-I/II. Protein expression values were normalised to β-actin. Percent control represents comparison with the respective vehicle-treated time-matched group (set as 100%). Values are means ± SEM of n = 4 (A) or n = 3 (B) experiments. Statistical analysis revealed no significant differences between SB202190-treated groups and time-matched vehicle controls.

Following incubation with SB202190 for 24 h, the conversion of LC3A/B-I into PE-conjugated LC3A/B-II was increased in a concentration-dependent manner (Figure 3A). LC3A/B-II levels attained a 2.9-fold and 3.2-fold increase with 5 μM and 10 μM SB202190, respectively (Figure 3A). Unconjugated LC3A/B-I protein was shown to become only slightly increased by SB202190, reaching a 1.7-fold induction after a 24-h incubation with 10 μM SB202190 (Figure 3A). Notably, following a 24-h incubation with the negative control SB202474, the level of PE-conjugated LC3A/B-II was not significantly increased (SB202474 at 10 μM, 120% ± 9.7% vs. vehicle control, 100% ± 8%, means ± SEM of n = 7). Time-course experiments revealed increased levels of PE-conjugated LC3A/B-II protein after a 6-h incubation with 10 μM SB202190 (1.7-fold vs. vehicle control) that peaked at 24 h (2.1-fold vs. vehicle control) (Figure 3B). After 48 h, protein levels of both LC3A/B-I and LC3A/B-II were increased as compared to the time-matched vehicle control.

### Inhibition of HO-1 activity decreases the anti-apoptotic effect of SB202190

**Figure 4:**
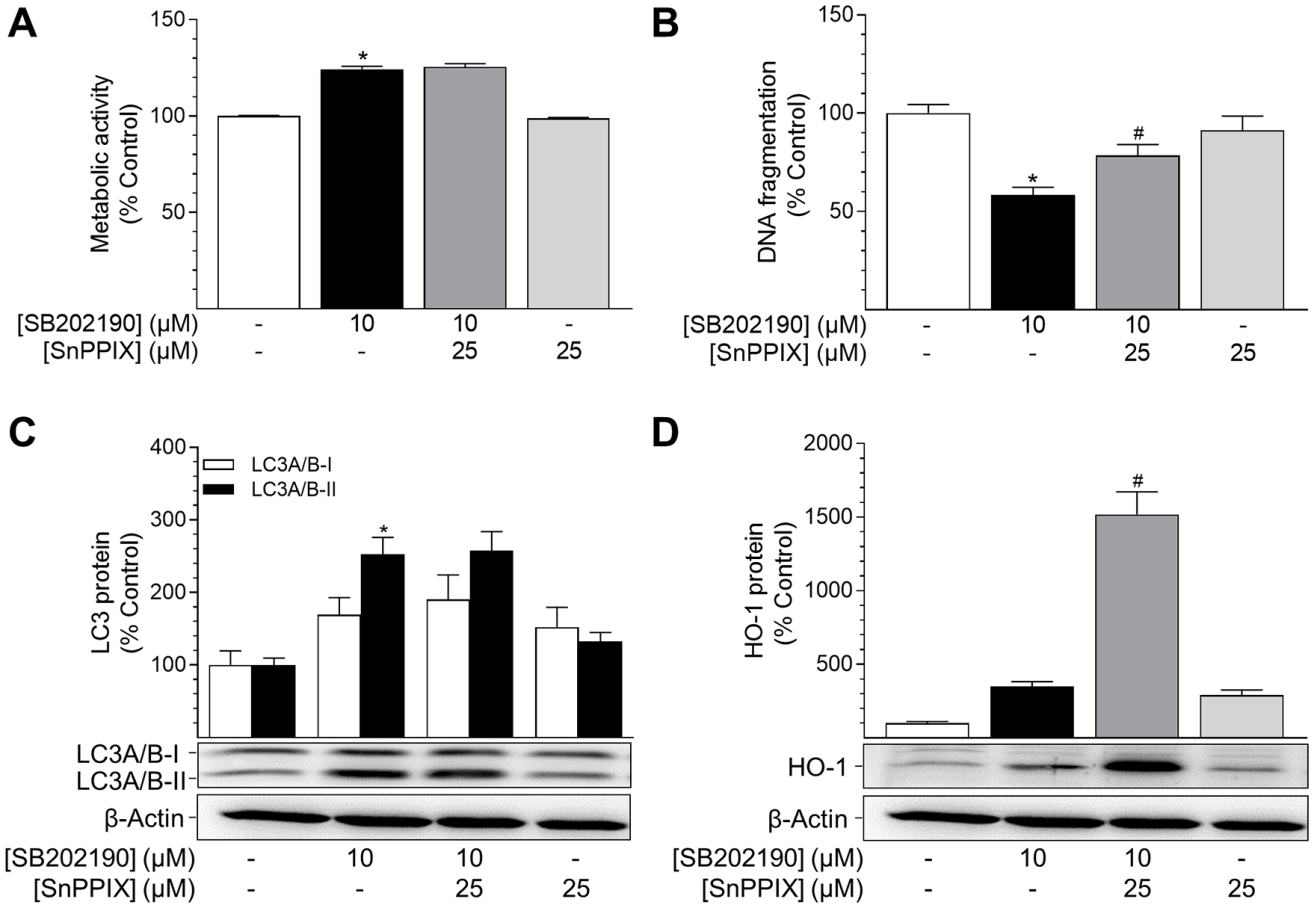
Impact of the HO-1 inhibitor SnPPIX on effects of SB202190 on metabolic activity, apoptosis and autophagy HUVEC were pre-incubated with the HO-1 activity inhibitor SnPPIX (25 μM) for 1 h followed by addition of SB202190 (10 μM) and continuation of incubation for another 24 h. Thereafter, cells were analysed for metabolic activity **(A)** and DNA fragmentation **(B)**. Lysates of cells were analysed for autophagy-related protein LC3A/B-I/II **(C)** and HO-1 **(D)**. Protein expression values were normalised to β-actin. Percent control represents comparison with vehicle-treated group (set as 100%). Values are means ± SEM of n = 21–24 (A), n = 10–12 (B), n = 12 (C) or n = 16 (D) experiments. ^*^P < 0.05 vs. vehicle control; ^#^*P* < 0.05 vs. SB202190; one-way ANOVA plus post hoc Bonferroni test.

To investigate the involvement of HO-1 in SB202190-mediated effects on autophagy and apoptosis, HO-1 activity was inhibited by the HO-1 inhibitor SnPPIX prior to and during stimulation with SB202190. Co-incubation with SnPPIX did not attenuate both, SB202190-mediated stimulation of metabolic activity (Figure 4A) as well as increase of PE-conjugated LC3A/B-II protein (Figure 4C). On the other hand, SnPPIX was shown to significantly reverse the SB202190-mediated anti-apoptotic effect in HUVEC (Figure 4B), although the SB202190-mediated increase of HO-1 protein level was further increased when cells were co-incubated with SnPPIX (Figure 4D). However, when applied to cells alone, SnPPIX did not significantly alter basal apoptosis in HUVEC (Figure 4B), albeit increasing HO-1 protein levels per se (Figure 4D).

### Inhibition of HO-1 protein expression decreases the anti-apoptotic effect of SB202190

**Figure 5:**
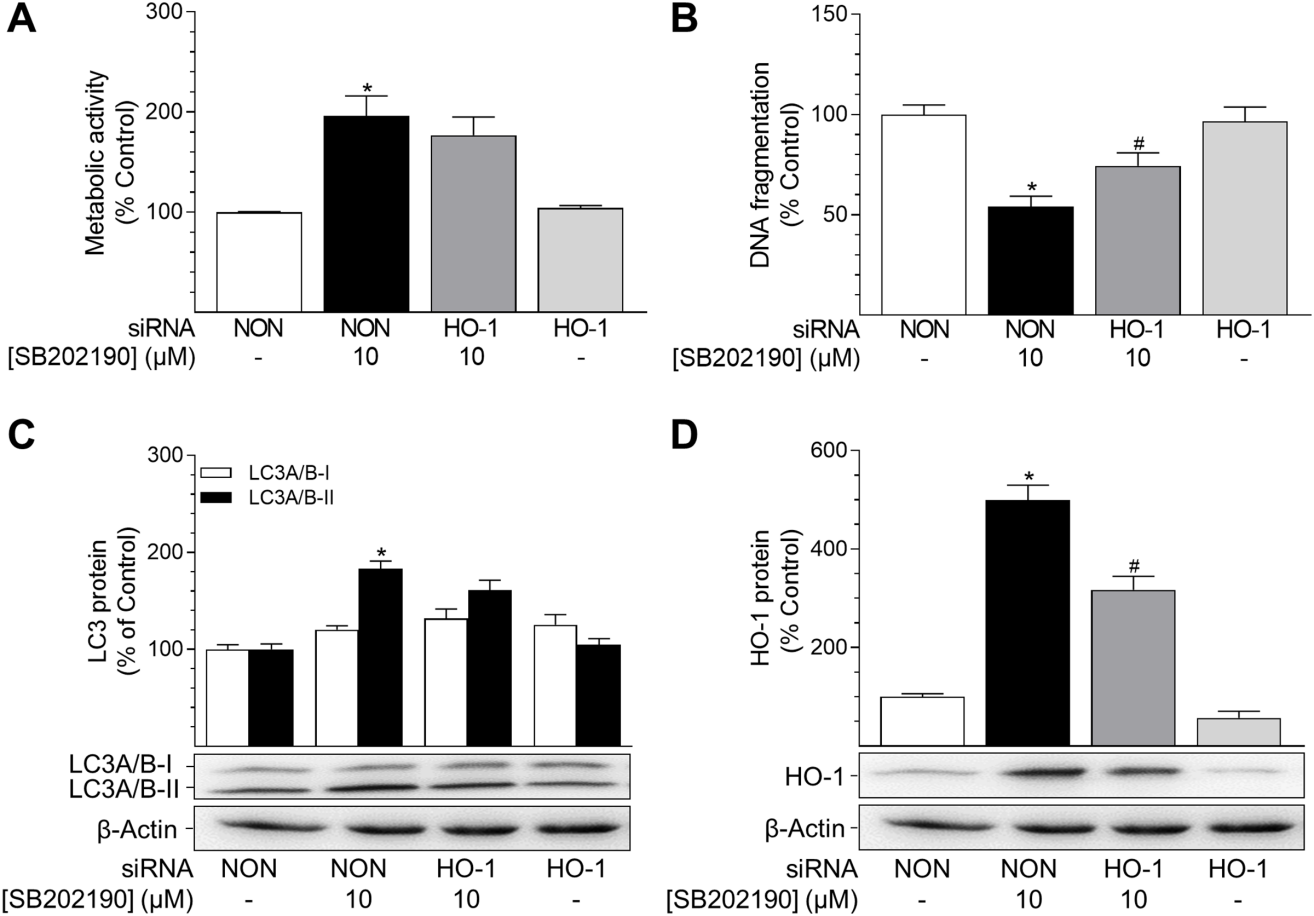
Impact of HO-1 siRNA on effects of SB202190 on metabolic activity, apoptosis and autophagy HUVEC were transfected with HO-1-specific siRNA or non-targeting siRNA (NON) 24 h prior to stimulation with SB202190 (10 μM). Following a 24-h incubation, cells were analysed for metabolic activity **(A)** and DNA fragmentation **(B)**. Lysates of cells were analysed for autophagy-related protein LC3A/B-I/II **(C)** and HO-1 **(D)**. Protein expression values were normalised to β-actin. Percent control represents comparison with vehicle-treated group (set as 100%). Values are means ± SEM of n = 13–16 (A), n = 18–20 (B) or n = 20 (C, D) experiments. ^*^P < 0.05 vs. vehicle control; ^#^*P* < 0.05 vs. SB202190; one-way ANOVA plus post hoc Bonferroni test.

To exclude possible unspecific effects of SnPPIX, additional experiments using siRNA interference were performed to knockdown HO-1 protein expression. In line with the results obtained when inhibiting HO-1 activity (Figure 4A), a knockdown of HO-1 did not alter the SB202190-mediated increase in metabolic activity (Figure 5A). Likewise, the SB202190-mediated increase of PE-conjugated LC3A/B-II protein level was not significantly altered by knockdown of HO-1 (Figure 5C). However, knockdown of HO-1 protein expression significantly reversed the SB202190-mediated decrease of DNA fragmentation in HUVEC (Figure 5B) without showing a pro-apoptotic action per se. SB202190-mediated HO-1 protein expression was significantly knocked-down by HO-1 siRNA (Figure 5D). Likewise, basal HO-1 protein expression was reduced by a comparable extent after transfection with HO-1 siRNA (Figure 5D). The respective expression rates were as follows: non-targeting siRNA, 100% ± 6%; non-targeting siRNA + SB202190 (10 μM), 499% ± 31%; HO-1 siRNA + SB202190 (10 μM), 317% ± 27%; HO-1 siRNA, 57% ± 13%, means ± SEM of n = 20 (Figure 5D).

### SB202190-mediated autophagy protects HUVEC from apoptosis

**Figure 6:**
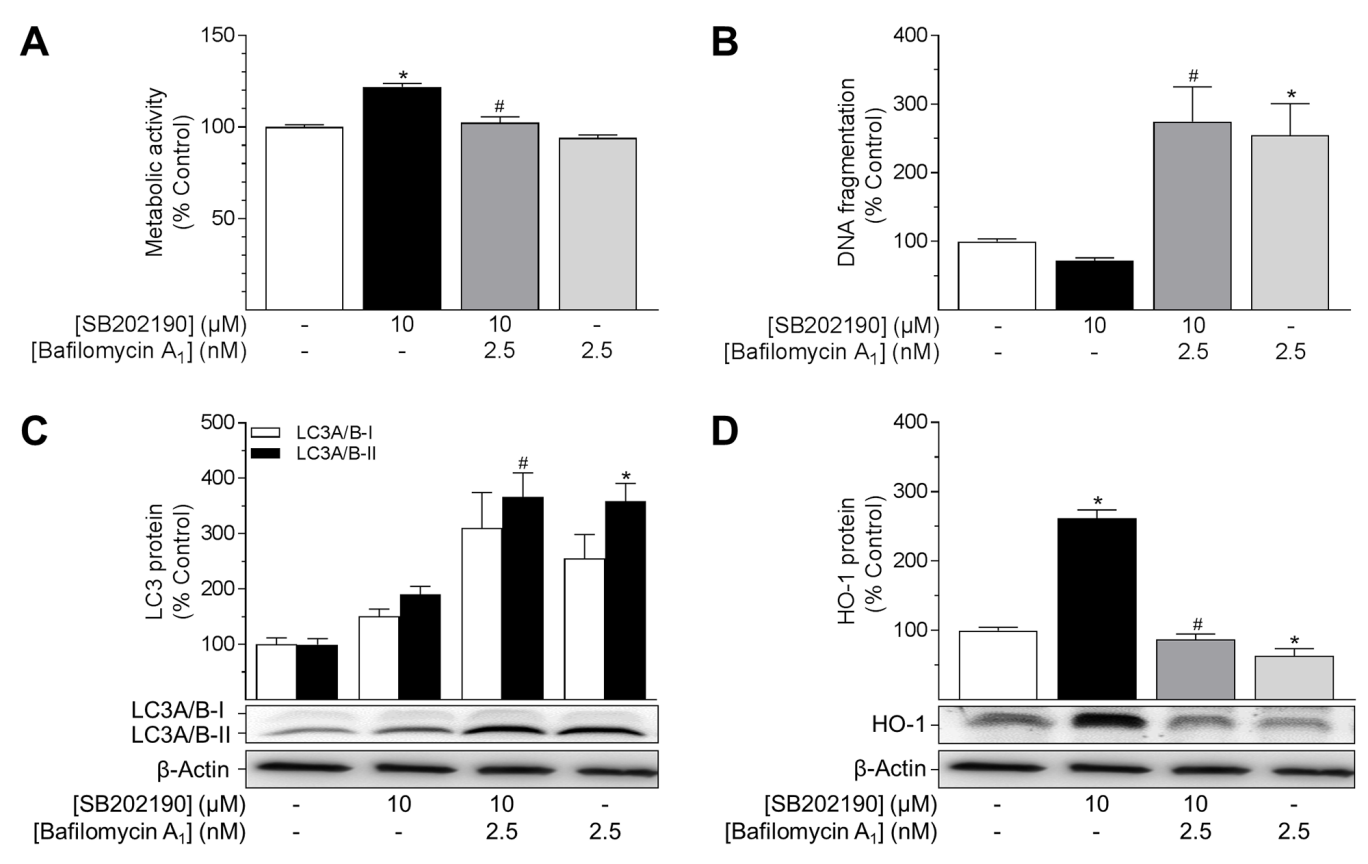
Impact of the autophagy inhibitor bafilomycin A_1_ on effects of SB202190 on metabolic activity, apoptosis, autophagy and HO-1 expression HUVEC were pre-incubated with the autophagy inhibitor bafilomycin A_1_ (2.5 nM) for 1 h followed by addition of SB202190 (10 μM) and continuation of incubation for another 24 h. Thereafter, cells were analysed for metabolic activity **(A)** and DNA fragmentation **(B)**. Lysates of cells were analysed for protein expression of LC3A/B-I/II **(C)** and HO-1 **(D)**. Protein expression values were normalised to β-actin. Percent control represents comparison with vehicle-treated group (set as 100%). Values are means ± SEM of n = 19–25 (A), n = 14–15 (B), n = 11 (C) and n = 12 (D) experiments. ^*^P < 0.05 vs. vehicle control, ^#^*P* < 0.05 vs. SB202190; one-way ANOVA plus post hoc Bonferroni test.

To further examine whether the cellular effects of SB202190 resulted from induction of autophagy, HUVEC were pre-treated with bafilomycin A_1_ (2.5 nM, 1 h), a late-phase inhibitor of autophagy, prior to incubation with SB202190. Bafilomycin A_1_ prevents maturation of autophagic vacuoles by inhibition of vacuolar H^+^-ATPase required for fusion of autophagosomes and lysosomes [44, 48]. Co-incubation with bafilomycin A_1_ fully reversed pro-metabolic (Figure 6A) and anti-apoptotic (Figure 6B) effects mediated by SB202190. Bafilomycin A_1_ alone explicitly increased DNA fragmentation under basal conditions (Figure 6B). Furthermore, bafilomycin A_1_ alone or in combination with SB202190 led to a considerable increase in LC3A/B-II protein levels due to inhibition of autophagosome formation and degradation (Figure 6C). In terms of HO-1, both SB202190-mediated as well as basal expression of HO-1 protein was profoundly decreased by co-incubation with bafilomycin A_1_ (Figure 6D).

## DISCUSSION

**Figure 7:**
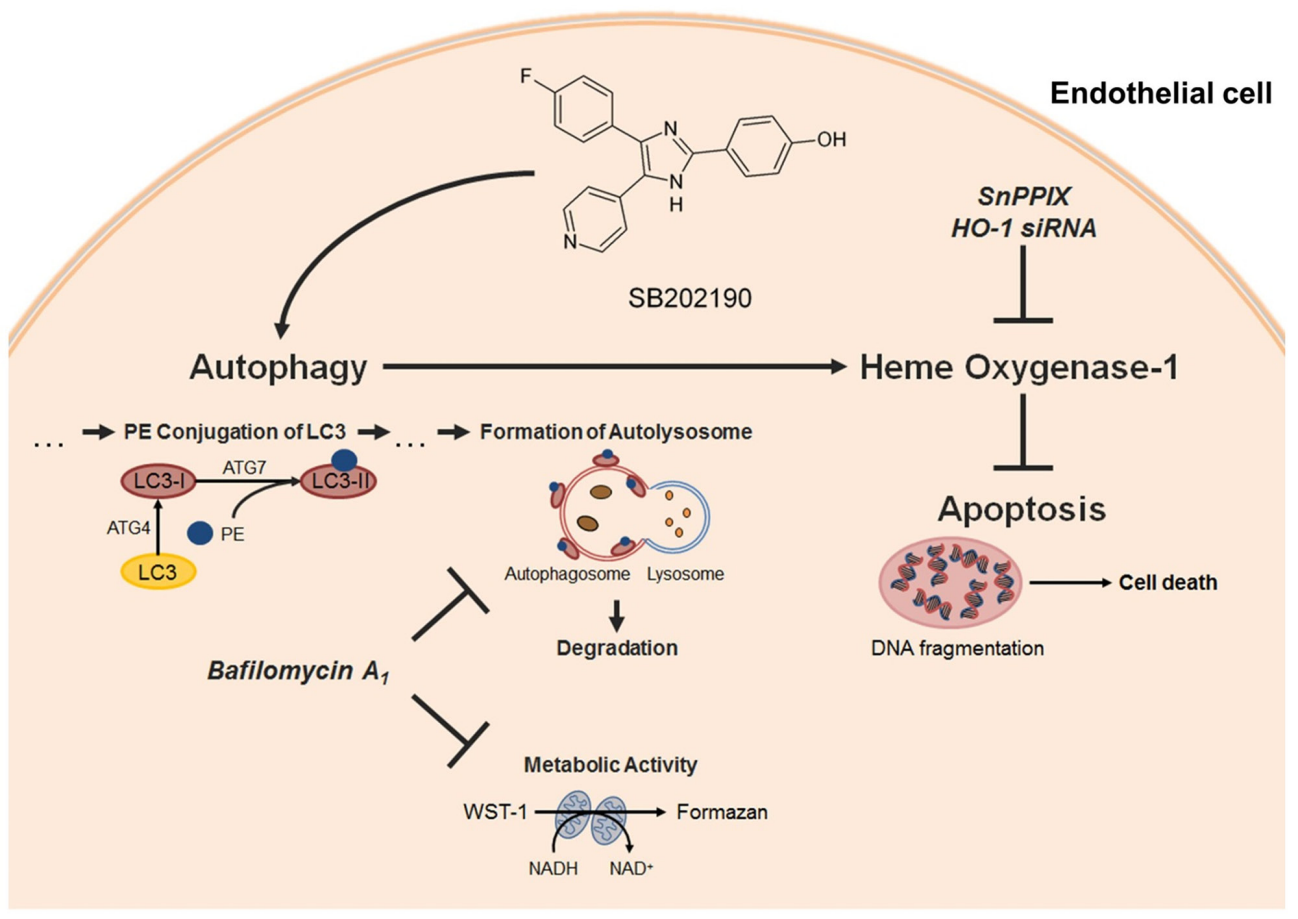
Proposed mechanism underlying the cytoprotective action of SB202190 in HUVEC The p38 MAPK inhibitor SB202190 inhibits apoptosis of endothelial cells by activation of autophagy followed by induction of the cytoprotective enzyme HO-1. Activation of autophagy was substantiated by increased metabolic activity and enhanced phosphatidyl ethanolamine (PE)-conjugation of LC3-I protein, resulting in LC3-II protein [43]. Inhibition or knockdown of HO-1 reversed anti-apoptotic effects, but not autophagy activation mediated by SB202190. Inhibition of autophagosome formation by late-phase autophagy inhibitor bafilomycin A_1_ [44, 48] reversed pro-metabolic effects and HO-1 induction by SB202190 thereby abolishing anti-apoptotic effects of the p38 MAPK inhibitor in HUVEC.

Atherosclerosis, induced by inflammation, accompanied by endothelial cell dysfunction and finally leading to apoptosis of endothelial cells, represents the main mechanism of adverse cardiovascular events [4]. Substances inhibiting endothelial cell apoptosis may therefore meet a therapeutic need to prevent the progression of vascular diseases. The present study demonstrates the p38 MAPK inhibitor SB202190 to decrease basal endothelial cell apoptosis. The underlying mechanism was shown to include both activation of autophagy and induction of the anti-oxidative enzyme HO-1, with autophagy posing an upstream event of HO-1 induction (Figure 7).

Initial experiments demonstrated two p38 MAPK inhibitors, SB202190 and SB203580, to induce the expression of HO-1 in HUVEC on both mRNA and protein level, whereas the inactive structural analogue SB202474 [12, 50] was virtually inactive in this respect. SB202190 and SB203580 have been previously reported to selectively inhibit p38α and p38β but not p38γ and p38δ isoform [22–26]. In line with our observations, a comparable modulation of HO-1 expression by p38 MAPK has been observed in other cellular systems. Accordingly, inhibition or genetic deficiency of p38α has been associated with an accumulation of reactive oxygen species (ROS) and upregulation of HO-1 that was mediated by the transcription factor nuclear factor (erythroid-derived 2)-like 2 (Nrf2) in mouse embryonic fibroblasts and murine RAW264.7 macrophages [36, 37]. Moreover, in primary cultures of rat hepatocytes, downregulation of HO-1 was demonstrated to occur via activation of p38α, p38β and p38δ, while upregulation was identified to be related to p38γ [51].

In our hands treatment of HUVEC with SB202190, but not with an inactive structural analogue without p38 MAPK-inhibitory activity, was associated with increased metabolic activity, a decreased rate of DNA fragmentation (marker of apoptosis) as well as an increased expression of PE-conjugated LC3-II protein, a marker of autophagy activation [47, 48]. These data are in line with studies showing SB203580-mediated inhibition of p38 MAPK to decrease tumour necrosis factor α-induced apoptosis in the human umbilical vein endothelial cell line EA.hy926 [27], starvation- and interleukin-1β-induced cell death of human pulmonary artery endothelial cells [28] and hydrogen peroxide-induced apoptosis of adult rat ventricular myocytes [29]. However, the functions of p38α and p38β have been discussed controversially dependent on cell type and stimulus. Thus, for HUVEC p38α and p38β were described to have a pro-apoptotic and an anti-apoptotic role, respectively [52]. In addition, p38β MAPK has been likewise shown to confer anti-apoptotic responses in HeLa [53] and rat mesangial cells [54] as well as in mice cardiac myositis [55]. Finally, another study indicated that p38 MAPK activation is not required for Fas-induced apoptosis in Jurkat cells [56].

The role of p38 MAPK in autophagy is likewise considered to be ambiguous and was reviewed in-depth recently [40]. For instance, a p38 MAPK-mediated autophagy has been associated with protection against dasatinib-induced hepatotoxicity [42]. Conversely, p38 MAPK blockade reversed senescence in primary human CD8^+^ T cells via induction of autophagy [39]. Moreover, there are some investigations suggesting p38 MAPK inhibitors to confer cell type-specific alterations of pro-autophagic gene expression via a p38 MAPK-independent mechanism resulting in defective autophagy [38, 41]. As outlined earlier, no alterations in autophagy were observed with a non-p38 MAPK inhibiting structural analogue in the present study, implying off-target effects of p38 MAPK inhibitors to occur in a cell type-dependent manner.

Our analyses with the p38 MAPK inhibitor SB202190 additionally revealed a concentration-dependent shift in cell cycle progression with arrest in the G_0_/G_1_ phase and significant reductions of both S phase and subG_1_ phase populations, indicating anti-proliferative as well as anti-apoptotic effects of the substance. In line with this finding, a modulation of cell cycle progression through HO-1 was reported by others [57–60]. Accordingly, overexpression of HO-1 was shown to mediate arrest of vascular cells in G_0_/G_1_ phase and inhibition of cell proliferation resulting in protection against vascular constriction [58, 59]. Li et al. further analysed the effect of CO, a product of HO-1, and noted that CO-mediated anti-proliferative effects in HUVEC and vascular smooth muscle cells were accompanied by inhibition of pro-apoptotic gene expression [60].

In the present study the involvement of enhanced HO-1 activity, resulting from increased protein expression, in the SB202190-mediated anti-apoptotic action was confirmed by experiments demonstrating that both HO-1 siRNA as well as the inhibitor of HO-1 activity, SnPPIX, significantly antagonised this response. Interestingly, co-administration of SnPPIX and SB202190 significantly increased HO-1 protein expression as compared to treatment with SB202190 alone. In accordance with these data, an increase of HO-1 protein expression after treatment with SnPPIX alone or in combination with other substances has been likewise reported by others [61–63]. In this context, SnPPIX was discussed to bind and remove transcriptional repressors of HO-1 gene expression just like its analogue substance heme, which is the natural inducer of HO transcription [63, 64]. However, despite of increased HO-1 protein levels, analysis of bilirubin formation clearly showed virtually complete inhibition of HO-1 activity by SnPPIX which was explained by SnPPIX’s competitive antagonism and blocking of the heme binding site of HO-1 [61–63].

Referring to the existing literature beneficial survival effects of HO-1 have been published in several other studies. For example, recent investigations reported activation of the Nrf2/HO-1 pathway to prevent hydrogen peroxide-induced damage in hepatocytes [65], to attenuate UVB-induced apoptosis in skin cells [66], to diminish gentamycin-induced toxicity in sensory hair cells [67] and to prevent high glucose-mediated endoplasmic reticulum stress-induced apoptosis in HUVEC [68]. It was further assumed that increased HO-1 expression leads to induction of anti-apoptotic genes and thus contributes to resistance to apoptosis [37, 69]. The essential anti-oxidative role of HO-1 was further emphasised by reports about a 6-year-old HO-1-deficient patient presenting, among other pathologies, enhanced endothelial cell injury and detachment of glomerular endothelium [70, 71].

In contrast to its role in the anti-apoptotic action of SB202190, a participation of HO-1 in SB202190-mediated increase in metabolic activity and induction of autophagy was excluded. Herein, protein expression of LC3-II, indicating autophagy activation, was not significantly altered by SnPPIX and HO-1 siRNA. As a matter of fact, some reports associated HO-1 with induction of autophagy which is a catabolic process of degradation of cellular material, e.g. long-lived proteins and organelles fulfilling key functions of cell survival in endothelial cells (reviewed in [44]). Investigating myocardial hypoxia-reoxygenation injury, Chen et al. reported HO-1 overexpression to induce autophagy resulting in protection of mitochondrial membrane stability and reduction of mitochondrial oxidation products [46]. HO-1-induced autophagy was further shown to reduce high glucose-induced apoptosis in mouse podocytes in an adenosine monophosphate-activated protein kinase (AMPK)-dependent manner [45]. However, since autophagy induction and enhancement of metabolic activity by SB202190 in HUVEC did not require HO-1, the exact mechanisms conferring these events have to be investigated in future studies.

On the basis of inhibitor experiments with bafilomycin A_1_, a late-phase inhibitor of autophagy, the present study further showed the SB202190-mediated autophagy process to represent the reason of increased metabolic activity and HO-1-mediated suppression of apoptosis. Accordingly, treatment of cells with bafilomycin A_1_ was demonstrated to significantly decrease both SB202190-mediated and basal expression of HO-1 protein along with normalising metabolic activity to the level of the respective vehicle control. Simultaneously, administration of low concentrations of the inhibitor dramatically increased apoptosis when co-incubated with SB202190 as well as when administered alone, indicating autophagy to be an essential basal survival mechanism in HUVEC. Hence, we suggest that SB202190-mediated induction of autophagy results in expression of HO-1 finally leading to the protective anti-apoptotic action. Although autophagy is predominantly described to depend on HO-1 [45, 72–77], there are also a few reports indicating a reversed mechanism. For example, Zhou et al. demonstrated cytoprotective autophagy in HeLa cells accompanied by induction of HO-1 [78]. In line with our study, inhibition of HO-1 did not attenuate autophagy induction, whereas inhibition of autophagy significantly blocked HO-1 upregulation and increased the rate of cell death [78]. It was further stated that flavonoid-induced autophagy likely contributes to activation of Nrf2, the main transcription factor of HO-1, thereby alleviating alcohol-triggered liver steatosis and inflammatory responses in mice [79]. Furthermore, low-dose ionising radiation-induced Nrf2-activation and subsequent HO-1 expression were attenuated by inhibition of autophagy or scavenging of ROS counteracting radio-resistance in the human lung adenocarcinoma cell line A549 [76].

Collectively, the present study demonstrates the p38 MAPK inhibitor SB202190 to activate the anti-oxidant enzyme HO-1 via an autophagy-dependent mechanism, finally leading to protection against basal endothelial cell apoptosis. Conversely, no evidence was obtained for a role of HO-1 in SB202190-mediated autophagy and enhancement of metabolic activity. The data thus provide novel mechanistic insights into the cytoprotective effect of p38 MAPK inhibition within the endothelium.

## MATERIALS AND METHODS

### Materials

SB202190 was obtained from Sigma-Aldrich (Taufkirchen, Germany). SB202474 and SB203580 were bought from Invitrogen (Darmstadt, Germany) and Tocris Bioscience (Wiesbaden, Germany), respectively. SnPPIX was from Enzo Life Sciences GmbH (Lörrach, Germany). SiRNA targeting HO-1 was purchased from Santa Cruz Biotechnology, Inc. (Heidelberg, Germany; sc-35554). Negative control siRNA was from Qiagen (Hilden, Germany; cat. no. 1022076). Lipofectamine™ RNAiMAX and OptiMEM were from Thermo Fisher Scientific Inc. (Schwerte, Germany). Bafilomycin A_1_ was bought from InvivoGen (Toulouse, France).

### Cell culture

Human umbilical vein endothelial cells (HUVEC) were purchased from Promocell (Heidelberg, Germany). Expansion, cell cultivation and experiments were performed using endothelial cell growth medium (ECGM) supplemented with 0.4% endothelial cell growth supplement (ECGS), 2% fetal calf serum (FCS), 0.1 ng/ml epidermal growth factor (EGF), 1 ng/ml basic fibroblast growth factor (bFGF), 90 μg/ml heparin and 1 μg/ml hydrocortisone (all from Promocell). HUVEC at passages 2 to 6 were grown in a humidified incubator at 37°C and 5% CO_2_ and used for experiments. HUVEC were seeded in the respective plates 24 h prior to medium change and stimulation. A 1-hour pre-incubation was performed in co-incubation experiments using SnPPIX or bafilomycin A_1_. All incubations were performed in ECGM. Most test substances were dissolved in DMSO and further diluted with ECGM yielding final DMSO concentrations in incubates of 0.02% (v/v) (for SB202190), 0.025% (v/v) (for bafilomycin A_1_) or 0.05% (v/v) (for SB202474 and SB203580). Vehicle for SnPPIX in incubates was ECGM containing 0.1% (v/v) NaOH (1 M). As vehicle control ECGM containing the respective amount of DMSO and/or NaOH was used. SiRNAs were diluted in RNase-free water according to the manufacturer’s instructions.

### Metabolic activity assay

The effect of test substances on the metabolic activity of HUVEC was analysed using WST-1 reagent (Roche Diagnostics, Mannheim, Germany). This assay is based on the bioreduction of the water-soluble tetrazolium salt WST-1 by NAD(P)H. Therefore, the metabolic activity of cells correlates directly to the amount of formazan dye formed. Briefly, HUVEC were seeded in 96-well plates at 10^4^ cells per well 24 h prior to incubation with test substances or vehicles. Following incubation, WST-1 reagent was added to cells in a final dilution of 1:10. In co-incubation experiments using SnPPIX, medium was refreshed prior to addition of WST-1 reagent to avoid influences in absorbance measurement due to the colouring of SnPPIX. Cells were further incubated for 30 to 90 min and absorbance was measured at 490/650 nm using an ELISA plate reader.

### DNA fragmentation ELISA

DNA fragmentation, an indicator of apoptosis, was determined using the Cell Death Detection ELISA^plus^ kit (Roche Diagnostics). The principle of the assay is the detection of cytoplasmic histone-associated DNA fragments generated during apoptotic cell death. Briefly, HUVEC were seeded in 6-well plates at 4 × 10^5^ cells per well prior to incubation with test substances or vehicles. Following incubation, floating cells were collected and combined with the adherent cells that were harvested by trypsinisation. After centrifugation (800 × g, 5 min) cells were resuspended in ECGM and counted using a haemocytometer. ELISA was performed according to the manufacturer’s instructions using 10^4^ cells per reaction.

### Transfection experiments

Knockdown of HO-1 protein expression was performed using Lipofectamine™ RNAiMAX transfection reagent according to the manufacturer’s instructions. Briefly, cells were seeded in 6-well plates at 2 × 10^5^ cells per well 24 h prior to transfection. For one well, transfection complexes were generated by mixing 45 pmol siRNA with 5 μl Lipofectamine™ RNAiMAX in OptiMEM to a final volume of 500 μl. Complexes were mixed thoroughly and incubated for 20–30 min at room temperature prior addition to cells. Supernatants were removed subsequently and cells were carefully covered with 500 μl complex solution before adding 2.5 ml of fresh ECGM. Control cells were transfected with a non-targeting siRNA (NON). Experimental settings were adapted for biochemical assays in 96-well plates. The final concentration of siRNA was 15 nM. After 24 h, transfection medium was replaced with fresh ECGM and cells were treated as stated above.

### Analysis of cell cycle distribution via flow cytometry

Cell cycle distribution of HUVEC was investigated by flow cytometry. In this assay, different stages of cell cycle can be distinguished by DNA content of cells quantifiable through DNA staining with propidium iodide. Due to cell cycle progression DNA content varies depending on cycle stage. Resting cells (G_0_/G_1_) have a DNA content of 1 which is increased during the synthesis phase (S phase). Cells with a DNA content of 2 are assigned to G_2_ and M phase. A DNA content < 1 is a result of apoptotic DNA degradation (subG_1_ population). Briefly, HUVEC were seeded in 6-well plates at 4 × 10^5^ cells per well prior to incubation with test substance or vehicle. Following incubation with different concentrations of SB202190 or its vehicle, floating cells were collected and combined with the adherent cells that were harvested by trypsinisation. Cells were pelleted by centrifugation (200 × g, 5 min) and washed twice with phosphate-buffered saline (PBS). Then, cells were resuspended in PBS to a density of 10^6^ cells/ml. For fixation and permeabilisation, cell suspension was adjusted to a content of 70% (v/v) ethanol by adding ice-cold absolute ethanol. Samples were incubated at 4°C for at least 1 h. Then, cells were centrifuged at 1000 × g for 5 min and resuspended in PBS to a cell density of 0.5 × 10^6^ cells/ml. RNA was digested with RNase A (0.1 mg/ml sample) for 20 min at 37°C. Subsequently, DNA was stained with propidium iodide (50 μg/ml sample) and 10^4^ cells per sample were analysed with an AccuriC6™ flow cytometer (BD Biosciences, Heidelberg, Germany). Discrimination between cell doublets and single cells was performed by analysing intersections of cell populations gated in FL2-H/FL2-A and FSC-H/FSC-A.

### Quantitative RT-PCR analysis

HUVEC were seeded in 24-well plates at 8 × 10^4^ cells per well prior to incubation with test substances or vehicles. Following incubation, total RNA was isolated using the RNeasy total RNA Kit (Qiagen). Expression levels of HO-1 and β-actin mRNA were determined by quantitative RT-PCR using the Applied Biosystems^®^ TaqMan^®^ RNA-to-C_T_™ 1-Step Kit (Thermo Fisher Scientific Inc.). Primers and probes for human β-actin and HO-1 were Applied Biosystems^®^ Gene Expression Assay™ products (Thermo Fisher Scientific Inc.). All experiments were performed according to the manufacturer’s instructions. HO-1 mRNA levels were normalised to β-actin and samples were compared to appropriate vehicle controls.

### Western blot analysis

For preparation of whole cell lysates, floating and adherent cells were collected and resuspended in 50 μl sample buffer (62.5 mM Tris/HCl, 2% [v/v] SDS, 10% [v/v] glycerol). Cells were then lysed by sonication, heated for 5 min at 95°C and centrifuged at 14,000 rpm for 5 min at 4°C. Supernatants were analysed for total protein concentrations using Pierce^®^ bicinchoninic acid (BCA) protein assay kit (Thermo Fisher Scientific Inc.) according to the manufacturer’s protocol. Samples of 50 μg protein containing 5% (v/v) β-mercaptoethanol were separated by 10% or 12% SDS-PAGE and transferred to nitrocellulose membrane by electroblotting using a semi-dry transfer system (Bio-Rad, Munich, Germany). Membranes were blocked for 1 h in 5% Blotting Grade Blocker (Bio-Rad) in Tris-buffered saline/Tween^®^ 20 (TBS-T) before incubation with specific primary antibodies at 4°C overnight. Antibodies directed against HO-1, LC3A/B and β-actin were obtained from Enzo Life Sciences GmbH, Cell Signaling Technology Europe (Leiden, The Netherlands) and Sigma-Aldrich, respectively. Afterwards, membranes were washed with TBS-T and probed with appropriate horseradish peroxidase-linked secondary antibodies. Secondary antibodies directed against mouse or rabbit IgG were purchased from Cell Signaling Technology Europe. Antibody binding was visualised by chemiluminescence and quantified by densitometric analysis using Quantity One 1-D Analysis Software (Bio-Rad). After analysis, membranes were stripped and reprobed. Expression of proteins was normalised to β-actin and compared to the appropriate vehicle controls.

### Statistics

Measurement values of DNA fragmentation and metabolic activity assays were analysed for statistical outliers in each experiment using Nalimov test. Flow cytometry data were analysed for identification of outliers using Grubbs test with alpha = 0.05 (GraphPad Prism 7.02 Software, San Diego, CA, USA). Statistical analysis was performed using GraphPad Prism 5.00 (GraphPad Software). One-way ANOVA plus post hoc Dunnett test was used for comparison of samples to vehicle control. Comparison among selected groups was carried out by Student’s two-tailed *t* test (for kinetics) or with one-way ANOVA plus post hoc Bonferroni test. All values are presented as mean ± standard error of the mean (SEM). A *P* value *P* < 0.05 was considered significant.

## Abbreviations

ECGM: endothelial cell growth medium
HO-1: heme oxygenase-1
HUVEC: human umbilical vein endothelial cells
LC3: microtubule-associated protein 1 light chain 3
MAPK: mitogen-activated protein kinase
Nrf2: nuclear factor (erythroid-derived 2)-like 2
PE: phosphatidyl ethanolamine
SB202190: 4-(4-Fluorophenyl)-2-(4-hydroxyphenyl)-5-(4-pyridyl)-1H-imidazole
SB202474: 4-Ethyl-2-(p-methoxyphenyl)-5-(4′-pyridyl)-1H-imidazole
SB203580: 4-(4-Fluorophenyl)-2-(4-methylsulfinylphenyl)-5-(4-pyridyl)-1H-imidazole
siRNA: small interfering RNA
SnPPIX: tin protoporphyrin IX
WST-1: 4-[3-(4-Iodophenyl)-2-(4-nitrophenyl)-2H-5-tetrazolio]-1.3-benzene disulfonate
